# Structural and Computational Study of the GroEL–Prion Protein Complex

**DOI:** 10.3390/biomedicines9111649

**Published:** 2021-11-09

**Authors:** Aleksandra A. Mamchur, Andrei V. Moiseenko, Irina S. Panina, Igor A. Yaroshevich, Sofia S. Kudryavtseva, Evgeny B. Pichkur, Olga S. Sokolova, Vladimir I. Muronetz, Tatiana B. Stanishneva-Konovalova

**Affiliations:** 1 Faculty of Biology, Lomonosov Moscow State University, 119991 Moscow, Russia; al.mam4ur@yandex.ru (A.A.M.); postmoiseenko@gmail.com (A.V.M.); iyapromo@gmail.com (I.A.Y.); sofiia.kudriavtceva@gmail.com (S.S.K.); sokolova@mail.bio.msu.ru (O.S.S.); 2 Shemyakin-Ovchinnikov Institute of Bioorganic Chemistry, Russian Academy of Sciences, 117997 Moscow, Russia; irinaspanina@gmail.com; 3 Faculty of Bioengineering and Bioinformatics, Lomonosov Moscow State University, 119991 Moscow, Russia; vimuronets@mail.ru; 4 National Research Center, Kurchatov Institute, 123098 Moscow, Russia; pichkur_eb@nrcki.ru; 5 Petersburg Nuclear Physics Institute Named by B.P. Konstantinov of NRC Kurchatov Institute, 188300 Gatchina, Russia; 6 Belozersky Institute of Physico-Chemical Biology, Lomonosov Moscow State University, 119991 Moscow, Russia

**Keywords:** molecular chaperones, GroEL, prion protein, Cryo-EM, molecular dynamics

## Abstract

The molecular chaperone GroEL is designed to promote protein folding and prevent aggregation. However, the interaction between GroEL and the prion protein, PrP^C^, could lead to pathogenic transformation of the latter to the aggregation-prone PrP^Sc^ form. Here, the molecular basis of the interactions in the GroEL–PrP complex is studied with cryo-EM and molecular dynamics approaches. The obtained cryo-EM structure shows PrP to be bound to several subunits of GroEL at the level of their apical domains. According to MD simulations, the disordered N-domain of PrP forms much more intermolecular contacts with GroEL. Upon binding to the GroEL, the N-domain of PrP begins to form short helices, while the C-domain of PrP exhibits a tendency to unfold its α2-helix. In the absence of the nucleotides in the system, these processes are manifested at the hundred nanoseconds to microsecond timescale.

## 1. Introduction

The prokaryotic GroEL/GroES complex is the most studied chaperonin from the heat shock proteins (HSP) family. It interacts with non-native conformations of various proteins, preventing their incorrect folding and aggregation in an ATP-dependent manner [1]. GroEL is an oligomeric protein complex consisting of 14 identical subunits combined into two rings of seven subunits. Each subunit of the GroEL heptamer ring consists of three domains: apical, intermediate, and equatorial. To perform its function, it interacts with its co-chaperonin GroES [2], which consists of seven identical subunits united in a dome-shaped ring structure. The apical domain of GroEL interacts with substrate proteins and GroES, while the equatorial one binds the ATP [3]. ATP’s binding and hydrolysis guide conformational changes of the complex, which result in the release of the folded substrate and ADP, forming the starting point for a new cycle. It is very probable that altering the physiological conditions may lead to a change in the steps of the functional cycle. For example, in high ADP concentration, the release of the ADP step is skipped [4]. Functional tests have indicated that the substrate-binding surface lies on the two α-helices (H and I) of the apical domain [5]. Co-crystallization of small hydrophobic peptides with GroEL revealed them to bind in an extended conformation in a groove between the H and I helices, forming contacts with the apical hydrophobic side chains [6,7]. Equatorial loops (residues 34–52) can also participate in substrate binding [8], as well as the mobile C-termini (a.a. 526–531), which extend from the equatorial domain into the ring cavity [9]. The introduction of mutations to these amino acids or the elimination of the whole region have shown to dramatically decrease the rate of substrate folding by GroEL [10,11,12]. Although there is no doubt as to the importance of the C-termini in GroEL function, experimental structural data are absent, due to the high mobility of the region.

Despite its protein-folding purpose, in some cases, GroEL may be involved in pathogenic protein transformation and aggregation, as in the case of the prion protein. Prions are protein infectious agents, causing neurodegenerative diseases called transmissible spongiform encephalopathies, such as scrapie, “mad cow disease”, fatal familial insomnia, kuru, Creutzfeldt––Jakob disease, and others. 

In mammalian cells, a normal isoform of the prion protein exists that is termed PrP^C^ (C-cellular). It is encoded by the highly conserved *Prnp* gene, consists of 253 amino acids, and is localized at the outer layer of the plasma membrane [13]. There are two domains in its tertiary structure: the unstructured N-terminal and the globular C-terminal, consisting of three α-helices and two β-layers. PrP’s N-terminal domain contains several regions: two charged clusters, an octarepeat region, and a hydrophobic domain [14]. The pathogenic isoform is termed PrPSc (Sc-scrapie, the first prion disease detected). Although PrPC and PrPSc have the same amino acid sequence, they are very different in their sec-ondary structure and biochemical properties. Most importantly, PrPSc contains more β-layers and can aggregate into fibrils [15,16,17].

It was previously suggested that the interaction of the infectious PrP with the bacterial chaperonin GroEL, which occurs in the gastrointestinal tract, is the key stage of the spongiform encephalopathies transmission [18]. The co-incubation of GroEL and PrP leads to the formation of protein aggregates, as demonstrated by the DLS turbidity study [18] and Immunoblot [19]. The presence of Mg–ATP was not required, but accelerated the reaction. Earlier studies suggested that chaperonin on its own cannot convert PrP^C^ to PrP^Sc^ without the initial presence of small amounts of PrPSc [20]. However, more recent studies have shown that this conversion is possible [18,21,22]. Interestingly, GroEL can also exhibit “anti-chaperone” activity: upon the interaction of GroEL with a denatured PrP, the resulting aggregates are more stable than upon the interaction with a structured PrP [21]. Biochemical and biophysical studies (using ELISA, DLS, fluorescence analysis, etc.) [23] demonstrated the inhibition of GroEL activity by a monomer of the ovine prion protein and its oligomeric forms. Thus, the action of GroEL on PrP can lead to the onset of a pathological process; however, the underlying mechanism is poorly understood, in particular due to the lack of structural information.

In this study, we present a cryo-EM structure of the initial GroEL–PrP complex and the results of molecular dynamics (MD) simulations to shed some light on the molecular basis of these interactions.

## 2. Materials and Methods

### 2.1. Purification of Chaperonin GroEL

*E. coli* cells (strain W3110) were transformed with the pOF39 plasmid that encodes GroEL and GroES. The cells were grown in LB medium in the presence of ampicillin (50 μg/mL). Extraction, sulfate ammonium fractionation, and DEAE–Sephacel ion-exchange chromatography were performed as described by Corrales and Fersht [24]. The proteins were eluted with a 0–500 mM NaCl gradient in a buffer containing 50 mM Tris, 2 mM DTT, and 0.1 mM EDTA, pH 7.2. GroEL was eluted at 0.33–0.38 M NaCl. The fractions containing GroEL were rapidly heated to 58 °C and then cooled to 25 °C; further, Mg^2+^–ATP (pH 7.0) was added to the final concentration of 2 mM and the solution was again incubated for 20 min at 58 °C. After that, GroEL was re-chromatographed on the DEAE–Sephacel under the same conditions. Pure fractions were concentrated using Centriprep centrifugal filters and then dialyzed against 10 mM of Tris–HCl buffer, pH 7.5. The obtained preparations of GroEL were stored in 80% ammonium sulfate at +4 °C. The purity of the GroEL sample was confirmed using SDS–PAGE (Figure S1). The concentration of GroEL_14_ was determined spectrophotometrically, considering that the molar extinction coefficient was 1.68 × 10^5^ M^−1^cm^−1^ [24].

### 2.2. Expression and Purification of the Ovine Prion Protein (PrP)

The VRQ (V136, R154, Q171) 23–234 a.a. (full-length amino acid sequence without the N-terminal signal peptide and C-terminal peptide, with one additional seryl residue on the N-terminus) variant of ovine PrP was expressed and purified following a procedure previously described in [25]. The VRQ variant was chosen as a more stable and compact variant.

BL21 (DE3) Escherichia coli strains (Novagen, Madison, WI, USA) transformed with the pET22b+ plasmids containing the PrP gene were cultivated in 500 mL of LB medium supplemented with 50 mg/mL ampicillin at 37 °C. Protein expression was induced by adding 1 mM IPTG to the medium, when OD600 reached 0.7. After PrP production, at night, cells were harvested by centrifugation. Then, cells were lysed in 50 mM Tris–HCl buffer, pH 8.0, containing a 0.5 mg/mL lysozyme, 10 mM EDTA, 0.1% Triton X-100, protease inhibitor cocktail for 30 min at 37 °C and sonicated with a Branson Digital Sonifer. Here, we collected insoluble inclusion bodies with PrP as a pellet. Inclusion bodies were solubilized at 6 M guanidine hydrochloride in 50 mM Tris–HCl buffer, pH 7.5, containing 0.5 M NaCl, during the night, and the suspension was applied to Ni2+-Chelating Sepharose fast flow in 20 mM Tris–HCl buffer, pH 7.4, containing 0.5 M NaCl, 8 M urea. At the next stage, Ni2+-bound PrP was renatured by the gradual replacement of the initial buffer with 20 mM Tris–HCl buffer, pH 7.4, containing 0.3 M NaCl and 20 mM imidazole. The prion protein was eluted with 1 M imidazole, pH 7.4, dialyzed against a large volume of 15 mM ammonium acetate buffer, pH 5.0, lyophilized, and stored at −20 °C. The purity of the sample was assessed with SDS–PAGE (Figure S2). Correct folding of PrP was confirmed with a circular dichroism spectrum (Figure S3).

### 2.3. Preparation of Cryo-EM Samples

Freeze-dried PrP was dissolved in 20 mM sodium acetate buffer, pH 4.0, and transferred into the 50 mM Tris–HCl buffer, pH 7.5, containing 1 mM EDTA (sample buffer-SB) by elution on a Sephadex–G25 desalting column. The GroEL ammonium sulfate suspension was centrifuged at 12,000× *g* for 10 min. The resulting pellet was dissolved in SB and then dialyzed against it for 2 hrs. Chaperonin activity was also tested by chaperone-dependent reactivation of denatured glyceraldehyde-3-phosphate dehydrogenase from rabbit muscle [23]. After that, 8 μM GroEL were co-incubated with 16 μM PrP in SB for 30 min at 21 °C. Formation of the GroEL–PrP complex was confirmed using dynamic light scattering (Figures S4 and S5). The obtained sample was centrifuged for 5 min at 13,000× *g* to precipitate the aggregates, and then the supernatant was concentrated two times on a Millipore Microcon with a filter of 100 kDa (from 50 to 25 μL). For grid preparation, 3 μL of the sample were applied to glow-discharged electron microscopy grids (copper Quantifoil R1.2/1.3), blotted for 3.0 sec and plunge-frozen in liquid ethane using the FEI Vitrobot Mark IV at 4.5° C and 100% RH.

### 2.4. Single-Particle Data Collection and Processing

1631 movies were collected using the Titan Krios electron microscope equipped with the Falcon II electron detector with a pixel size of 1.107Å. Per exposure, 25 frames were recorded with the dose of 4.0 e/Å^2^ per movie frame in the defocus range from −1.0 to −2.6 μm. Motion correction and CTF estimation were performed with MotionCor2 and Ctffind, and particles were picked with CrYOLO [26]. After two rounds of 2D classification in Relion [27], 106 k particles were selected for initial model building and 3D classifications. Additional 2D classification was performed with GPU ISAC [28] to assess the heterogeneity of the sample (Figure S6, Video S1). 3D classifications in Relion allowed the selection of the particle subset that exhibits some additional density in the GroEL cavity, however, is visible only at low map thresholds. We applied the C2 symmetry expansion to merge the information from both GroEL rings together, thus effectively doubling the number of particles present in the reconstruction. This 3D classification using a mask that covers one of the GroEL rings with its central cavity allowed for separation of the classes with a clear density between the apical domains (Figure S7). The particle density outside the mask was subtracted to decrease its influence on the classification. The particles from the density-containing classes were subjected to final 3D refinement, providing the density map with a 4.0Å resolution, the local resolution was estimated with PHENIX local_resolution (Figures S8 and S9, Table S1).

### 2.5. Molecular Dynamics

Starting conformations of GroEL and PrP were chosen from a molecular dynamics simulation of two separated proteins, conducted by our group earlier [29,30]. The GroEL–PrP complex was assembled in two variants: GroEL–PrP(N) with the N-domain in the GroEL cavity and GroEL–PrP(C) with the C-domain in the GroEL cavity. The starting orientation and insertion depth of PrP(N) or PrP(C) within the GroEL cavity were chosen so as to immerse the PrP domain to the maximum depth with the minimum intersection of atoms. Both variants were aligned with cryo-EM density. For details, see pdb files of the starting structures deposited to the Zenodo archive (10.5281/zenodo.5590094).

Molecular dynamics simulations were carried out in the GROMACS software package [31] version 2020.1 using the a99SB-disp force field, which was developed to capture the dynamics of both folded and disordered proteins [32]. The integration time step of 2 fs was used and 3D periodic boundary conditions were imposed. Simulations were performed at 300 K temperature and 1 bar pressure constants using the V-rescale [33] and the Parrinello–Rahman [34] algorithms, respectively. A 12 Å cutoff radius was defined for the Coulombic and van der Waals interactions. Electrostatic effects were treated using the particle-mesh Ewald summation [35]. An explicit solvent model was used (TIP4P-D [36]), the ionic strength of the solution corresponded to 150 mM NaCl. Before MD simulations, the systems were subjected to energy minimization (1000 conjugate gradient steps), followed by heating from 5 to 300 K for 5 ns. Protein and solvent molecules were coupled separately. For each variant of the GroEL–PrP complex, 500 ns trajectories were received. Supplementary Videos S2 and S3 demonstrating the course of MD trajectories with GroEL–PrP(N) and GroEL–PrP(C) complexes are available in the Zenodo archive (10.5281/zenodo.5590094).

MD data analysis was performed with the MDAnalysis Python package (version 2.0.0, NumFOCUS, Austin, TX, USA) [37,38].

## 3. Results

### 3.1. Cryo-EM Structure of the GroEL–PrP Complex

**Figure 1:**
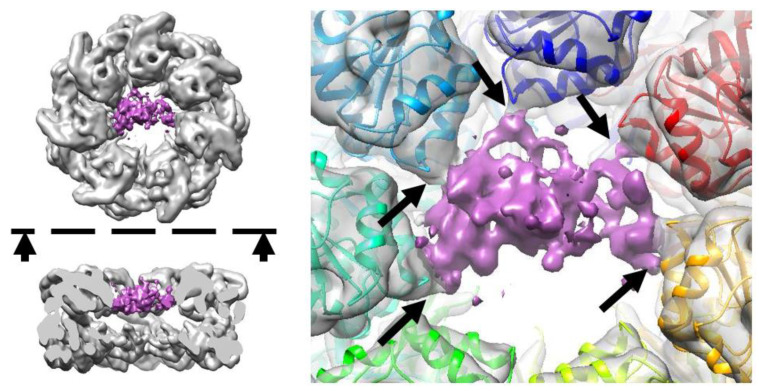
Density map indicating the formation of the GroEL–PrP complex. The black arrows on the right panel point to the contacts between the PrP and GroEL I helices. The dashed line and arrows on the left panel indicate the direction of the side view cross-section.

The obtained cryo-EM map (Figure 1) contains an additional density in the apical domain region, in comparison to the apo GroEL structures. This additional density is attributed to Prp and its contacts with the I helices of the GroEL apical domains. The signal-to-noise ratio of the density is strong enough to confirm the binding of the PrP to the GroEL, which happens in the sample that does not contain ATP. However, the local resolution of the obtained map does not allow to resolve the PrP secondary structure. The resolution of the PrP map region appears to be low, due to the strong conformational heterogeneity and mobility of the PrP. Insights into mobility can be obtained from ISAC 2D ordered class averages (Video S1).

### 3.2. Correlation between Cryo-EM and MD Simulations

The result of cryo-EM shows the structure averaged over the ensemble of particles, while the result of MD simulations gives the structure of a single molecular complex averaged over the time. Since the MD approach allows us to simulate the time evolution of the GroEL–PrP system, the validity of the result is determined by whether the simulation time is sufficient for the system to exhibit equilibrium behavior. To determine the sufficiency, we compared the cryo-EM local resolution map (Figure S9) with the probability distribution map (described in [30]) obtained from MD by averaging the coordinates through the time steps (Figure S10). These maps exhibit the same features. First, GroEL conformational diversity in the ensemble rises in the sequence equatorial–intermediate–apical domain. Second, due to the conformational diversity of the complex, the structure of the full-length PrP cannot be seen: at the appropriate (for the GroEL) threshold, the corresponding PrP density only contains a portion of about 1/3 of its volume. Additionally, one can notice that GroEL apical domains in contact with PrP are better resolved in the regions facing inward towards the cavity than those belonging to the non-contacting subunits. This is expected, because the apical domain being in direct contact with PrP should have lower entropy, both thermodynamic as well as conformational. This feature is manifested both in cryo-EM and MD maps. The lack of contradictions between cryo-EM and MD results supports our belief in the validity of theoretical data. Thus, it allows us to support the obtained low-resolution data for the GroEL–PrP complex with some atomic-level observables from MD.

### 3.3. Analysis of MD Simulation of the GroEL–PrP Complex

**Figure 2:**
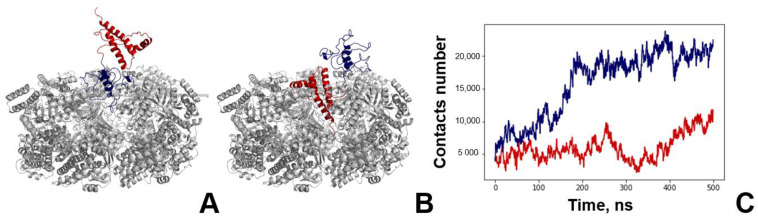
Molecular dynamics of GroEL–PrP complex formation. (**A**) Starting conformation of the GroEL–PrP(N) complex; grey—GroEL, blue—PrP N-terminal domain, red—PrP C-terminal domain. (**B**) Starting conformation of the GroEL–PrP(C) complex; grey—GroEL, blue—PrP N-terminal domain, red—PrP C-terminal domain. (**C**) Dynamics of GroEL–PrP(N) (blue) and GroEL–PrP(C) (red) number of contacts during the molecular simulations trajectory.

The main concern for the modelling of any molecular complex with PrP is the choice of its full-length structure. The problem lies within its N-terminal domain, which is an intrinsically disordered region. Previously, we used a two-step approach consisting of de novo bioinformatic modelling and subsequent molecular dynamics (Figure S11) to show that, although there is no stable conformation for the PrP N-domain, it forms a compact coil and contains characteristic contacts within [29]. In this work, we used the typical structure of the full length PrP to simulate its complex with GroEL. We also used contact analysis to study the GroEL–PrP complex formation. The contact criteria are based on a 0.7 nm cut-off. For a clearer understanding of all of the figures below, blue elements relate to the GroEL–PrP(N) complex (Figure 2A) and red-to the GroEL–PrP(C) (Figure 2B).

The number of GroEL–PrP contacts increases along the MD trajectories (Figure 2C), which, apparently, corresponds to the maturation of the complex. Several distinguishing features can be observed for the GroEL–PrP(N) and GroEL–PrP(C) cases. Firstly, the total number of contacts in the GroEL–PrP(N) complex is two times higher than in the GroEL–PrP(C). Secondly, the number of contacts for the GroEL–PrP(C) fluctuates along the whole trajectory, while in the case of GroEL–PrP(N) the fluctuations are reduced after 200 ns. Also, in the GroEL–PrP(N) sample, there are five closely connected subunits of GroEL (A, C, D, F, G), while in the GroEL–PrP(C) sample, only two such subunits are present (A, F) (Table 1). A detailed analysis on the contacts between PrP and each subunit of GroEL is provided in the supplementary information (Figures S12 and S13). These observations may indicate that the GroEL–PrP(N) complex is more energetically favorable than the GroEL–PrP(C).

### 3.4. GroEL Conformation Dynamics

**Figure 3:**
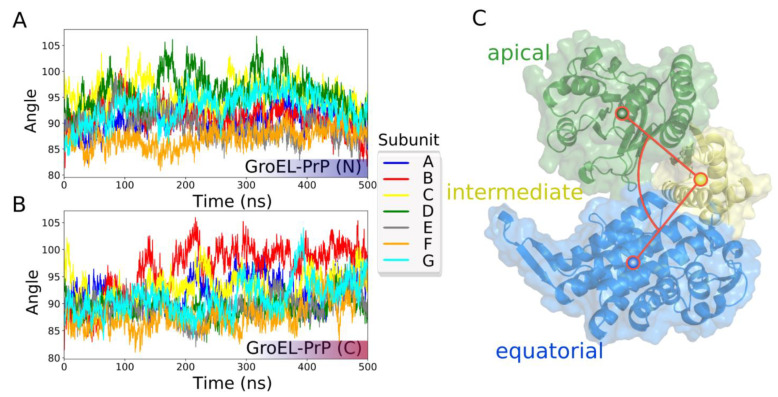
GroEL subunits mobility in terms of the angle between the centers of mass of the domains. (**A**) GroEL–PrP(N) trajectory. (**B**) GroEL–PrP(C) trajectory. (**C**) Illustration and surface representation of the domains: green—apical; yellow—intermediate; blue—equatorial.

To analyze the behavior of the chaperonin during the trajectory, we studied the mutual arrangement of the domains of each GroEL subunit along with GroEL subunit’s radius of gyration (Rg). The results of this analysis are shown in Figure 3A and Figure S14.

Initially, all subunits have slightly different conformations (they are not absolutely symmetric), because the starting conformations are themselves the result of molecular dynamics simulations (see materials and methods).

As one can see in Figure 3A and Figure S14, each of the GroEL subunits of the GroEL–PrP(N) complex show similar conformations at the beginning and the end of the trajectory. At the same time, this effect is not observed in the GroEL–PrP(C) complex.

### 3.5. PrP Conformation Dynamics

**Figure 4:**
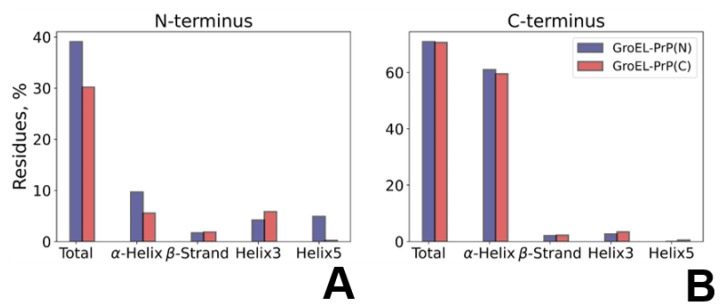
PrP’s secondary structure changes upon binding to GroEL. The main secondary structure component’s lifetime in the N-terminal (**A**) and C-terminal (**B**) domains of PrP revealed within the GroEL–PrP(N) (blue) and GroEL–PrP(C) (red) trajectories; The bar indicates the trajectory percentage (averaged over MD) of amino acid residues in the secondary elements of PrP: the total percentage sum, including residues in turns and bends, along with the main structure elements shown separately.

To analyze the behavior of the PrP along the trajectory, we performed a quantitative secondary structure analysis. The results are shown in Figure 4A, Figures S15 and S16. Starting from the same model structure, PrP undergoes varying conformational changes in GroEL–PrP(N) and GroEL–PrP(C) complexes. The data (Figure 4) reveal the relative percentage changes occurring for each secondary structure component calculated for the PrP domain being bound to GroEL or exposed into solution. The binding of the disordered N-terminal PrP domain to GroEL results in a more ordered secondary structure, as compared to the unbound N-domain in the GroEL–PrP(C) complex (Figure 4A). Conversely, the PrP C-terminus being placed inside the GroEL cavity tends to partially lose its secondary structure (Figure 4B). The slight total decrease of the GroEL-bound C-terminus secondary structure includes the loss of a fragment (residues 172–178) of a stable helix H2 (Figure S16), which is not observed within the dynamics of the GroEL–PrP(N) complex.

## 4. Discussion

**Figure 5:**
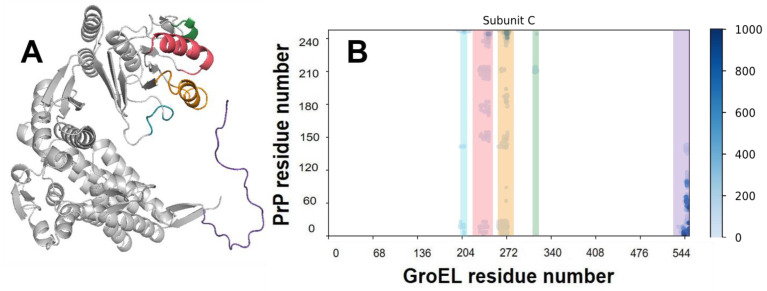
Regions of GroEL contacting with PrP. (**A**) Model of the GroEL subunit with colored contacting regions. Cyan—residues 202–207, coral—residues 227–244, orange—residues 254–272, green—residues 309–312, purple—C-tail. (**B**) Example of the GroEL–PrP contact matrix (produced for Subunit C and PrP of the GroEL–PrP(N) complex). The abscissa shows the number of GroEL residues, the ordinate shows the number of PrP residues. Contacts between atoms are marked with dots, the color of which depends on the time of existence of the contact. Contacting regions of GroEL are lighted with the same colors as in (**A**).

According to our MD, any region of PrP could potentially form contacts with GroEL (Figures S12 and S13). However, the hydrophobic domain of the N-terminal part of PrP (residues #110–124) shows the highest frequency of occurrence on the contact map. Indeed, our results may indicate that the disordered N-domain of PrP forms an energetically more favorable complex with GroEL than its C-domain (Figure 5, Table 1). Thus, it can be assumed that the particle ensemble collected with cryo-EM (Figure 1) mostly corresponds to the GroEL–PrP(N) complex.

According to contact matrices (Figures S12 and S13), there are five regions in a GroEL subunit that form contacts with PrP (Figure 5): two unstructured loops (residues #202–207, 309–312), helices H (227–244) and I (254–272), and flexible C-tail (530–546). All these contact regions correlate with literature data and confirm the role of H and I helices and C-tails in substrate binding.

Importantly, we demonstrated that, being immersed in the GroEL cavity, the N-terminus of PrP exhibits an upshifted tendency to form helix-like regions (Figure S15). These small helices, appearing at random positions due to the interaction between the PrP’s N-domain and GroEL, expose the appetite of chaperonin for the folding of disordered regions, even in the absence of ATP. The GroEL–PrP(C) complex exhibits a contrary tendency. The portion of the secondary structures for the C-domain is slightly reduced upon its interaction with GroEL (Figure 4B). According to our MD simulations, this reduction transpires because of the unweaving of the N-terminus of the H2-helix (residues #170–180) (Figure S16). This observation can be attributed to the refolding action of GroEL, which implies the disruption of misfolded regions. Additionally, once more, the tendency for that action could be seen without ATP in the system.

Our results suggest the following mechanism of the transformation of the PrP^C^ to the PrP^Sc^ facilitated by GroEL. The GroEL interacts with both disordered N-terminal and globular C-terminal domains of PrP in the early molecular complex, even without nucleotides or co-chaperones in the system. The unfolding of the globular C-domain of PrP by GroEL could lead to the reassembly of the PrP^C^ helices to the amyloid strands, but the actual rate of that process is controlled by the negligible number of formed GroEL–PrP(C) particles because of the high prevalence of the GroEL–PrP(N). We could make a heuristic forecast that without the N-domain or with changes within the N-domain, which decrease the interaction energy in the GroEL–PrP(N) complex, the actual rate of the PrP^C^ to PrP^Sc^ transformation in the presence of GroEL will increase. The verification of this prognosis requires further experimental studies of the PrPC conversion in the presence of GroEL.

## Figures and Tables

**Table 1:** Number of contacts between GroEL and PrP in GroEL–PrP(N) and GroEL–PrP(C) samples.

**GroEL–PrP(N)**								
**Subunit\Time, ns**	**A**	**B**	**C**	**D**	**E**	**F**	**G**	**Sum**
**0**	1452	408	444	0	20	298	1333	**3955**
**50**	2442	142	1535	555	64	0	2642	**7380**
**100**	3270	15	2222	1011	1563	459	1527	**10,067**
**150**	2427	962	2667	1673	705	200	3082	**11,716**
**200**	3454	80	5675	2344	381	3155	2743	**17,832**
**250**	1974	9	5977	3479	744	2826	3179	**18,188**
**300**	2738	129	4480	2620	798	4092	1867	**16,724**
**350**	2465	864	5790	3179	680	3646	3087	**19,711**
**400**	1970	425	7387	2319	610	3832	4370	**20,913**
**450**	2410	277	6781	4137	524	2989	2835	**19,953**
**500**	1880	444	8282	4240	487	2966	4125	**22,424**
**average**	**2407**	**341**	**4658**	**2323**	**598**	**2224**	**2799**	
**GroEL–PrP(C)**								
**Subunit\Time, ns**	**A**	**B**	**C**	**D**	**E**	**F**	**G**	**Sum**
**0**	1955	319	883	0	13	780	456	**4406**
**50**	1427	43	631	0	2	1166	448	**3717**
**100**	1896	0	32	0	0	90	1117	**3135**
**150**	4133	123	31	0	1	52	92	**4432**
**200**	4140	33	8	0	19	359	94	**4653**
**250**	3649	593	2000	114	637	683	166	**7842**
**300**	2852	373	9	0	55	722	270	**4281**
**350**	1713	78	3	0	122	1917	469	**4302**
**400**	1480	62	3	0	220	2262	2474	**6501**
**450**	3634	0	125	0	678	2417	1759	**8613**
**500**	4345	0	893	0	577	4788	1172	**11,775**
**average**	**2839**	**148**	**420**	**10**	**211**	**1385**	**774**	

## Data Availability

The data presented in this study are openly available in the Zenodo archive (10.5281/zenodo.5590094): the pdb files of the starting structures of GroEL-PrP(N) and GroEL-PrP(C) complexes (GroEL-PrP_N_start.pdb, GroEL-PrP_C_start.pdb), ISAC 2d classes showing the mobility of the PrP inside the GroEL cavity (Supplementary Video S1) and the trajectory movies of both simulations (Supplementary Videos S2 and S3). The cryo-EM map was deposited to EMDB with ID EMD-13762.

## Supplementary Materials

The following are available online at https://www.mdpi.com/article/10.3390/biomedicines9111649/s1, Figure S1. SDS-PAGE of samples obtained at the last stage of GroEL purification on DEAE-Sephacel. 1—GroEL standard solution; 2-15—fractions collected during second chromatography. The arrow shows the band corresponding to the prion protein. Figure S2. SDS-PAGE of samples obtained during the purification of the prion protein carried out on the Ni2+-Chelating Sepharose fast flow. 1—protein molecular weight marker; 2–6—fractions obtained by affinity chromatography. Molecular weight markers are indicated on the left. The arrow shows the band corresponding to the prion protein. Figure S3. Far-UV CD spectra of the recombinant VRQ (V136, R154, Q171) variant of ovine PrP (23–234) at 20 °C. [θ]MRW, mean residue ellipticity (deg × cm^2^/dmol of residue). Figure S4. Hydrodynamic diameter of particles detected after co-incubation of GroEL (8 μM) with PrP monomers (16 μM) in 50 mM Tris-HCl buffer, pH 7.5 containing 1 mM EDTA. The populations of particles that are complexes are in gray, and the control of individual chaperonin complexes (dotted line) and PrP monomers (solid line) are shown separately on the plots. (a) 0 min of co-incubation; (b) 10 min of co-incubation; (c) 30 min of co-incubation; (d) 50 min of co-incubation; (e) 120 min of co-incubation. Figure S5. Hydrodynamic diameter of PrP molecules in 50 mM Tris-HCl buffer, pH 7.5 containing 1 mM EDTA before (solid line) and after (dotted line) incubation at 21 °C for 2 h. Figure S6. ISAC 2d class averages. Top view class averages #75-104 (2nd to 4th rows from the top) exhibit an additional density in the central cavity. Figure S7. Single particle processing workflow. Number of particles in each class is indicated. Figure S8. FSC curves for the symmetry-expanded reconstruction provide the 4A resolution based on 0.143 criterion. (Green—unmasked half-maps, blue—masked half-maps, red—phase randomized masked half-maps, black—corrected). Figure S9. GroEL-PrP cryo-EM local resolution map. Figure S10. Heavy atoms distribution combined for both GroEL-PrP(N) and GroEL-PrP(C) MD simulations projected on the Cryo-EM density map. Figure S11. Workflow for the full-length PrP model creation. Red cartoon+surface is the representation of the globular C-domain, varicoloured coils on the left represent top-scored solutions of de novo modeling for the N-domain in Alpfafold II [Jumper J. et al. Highly accurate protein structure prediction with AlphaFold //Nature.—2021.–T. 596.—№. 7873.—C. 583–589.], blue cartoon+surface on the right represents the common structure of the compact N-domain obtained after subsequent molecular dynamics and a cluster analysis of its conformations. G123-G124 are the chosen residues to separate the N- and C-domains in the representation. Figure S13. Contact matrices and dynamics of the number of contacts of the GroEL-PrP(C) complex by GroEL subunits. Figure S14. GroEL subunits mobility in terms of the radius of gyration (Rg): GroEL-PrP(N) trajectory (left); GroEL-PrP(C) trajectory (right). Rg of each subunit was calculated separately. Figure S15. Dynamics of the PrP secondary structure along the GroEL-PrP(N) (left) and GroEL-PrP(C) (right) trajectories. Figure S16. Presence of alpha-helices in the PrP structure across the calculated trajectory. Table S1: Data collection and processing statistics. Video S1: ISAC 2d classes showing the mobility of the PrP inside the GroEL cavity. Video S2: The course of MD trajectories with the GroEL-PrP(N) complex. Proteins are shown with tube representation using VMD program (version 1.9.4a12): grey—GroEL, blue—PrP N-terminal domain, red—PrP C-terminal domain. Only the Cα atoms of both proteins are shown for clarity. The trajectory is shown with a 250 ps timestep, with a smoothing window size of 10 for GroEL and 5 for PrP. Video S3: The course of MD trajectories with the GroEL-PrP(C) complex. Proteins are shown with tube representation using the VMD program (version 1.9.4a12): grey—GroEL, blue—PrP N-terminal domain, red—PrP C-terminal domain. Only the Cα atoms of both proteins are shown for clarity. The trajectory is shown with a 250 ps timestep, with a smoothing window size of 10 for GroEL and 5 for PrP. Videos S1–S3 are available in the Zenedo archive (10.5281/zenodo.5590094).
