# Supplementary material for: Structural and Computational Study of the GroEL–Prion Protein Complex
Source: Biomedicines. 2021 Nov 9;9(11):1649. doi: 10.3390/biomedicines9111649 (PMC8615626; doi:10.3390/biomedicines9111649)
Supplement: Supplementary file 1 [file biomedicines-09-01649-s001.zip › biomedicines-1421495-supplementary.pdf]

Structural and Computational Study of the GroEL - Prion Protein complex

Aleksandra A. Mamchur[1,7], Andrei V. Moiseenko[1,7], Irina S. Panina[2], Igor A. Yaroshevich[1], Sofia S. Kudryavtseva[1,3], Evgeny B. Pichkur[4,5], Olga S. Sokolova[1], Vladimir I. Muronetz[3,6], Tatiana B. Stanishneva-Konovalova[1*]

[1] Faculty of Biology, Lomonosov Moscow State University, Moscow, Russia
[2] Shemyakin-Ovchinnikov Institute of Bioorganic Chemistry, Russian Academy of Sciences, Moscow, Russia
[3] Faculty of Bioengineering and Bioinformatics, Lomonosov Moscow State University, Moscow, Russia
[4] National Research Center «Kurchatov Institute», Moscow, Russia
[5] Petersburg Nuclear Physics Institute Named by B.P. Konstantinov of NRC «Kurchatov Institute», 1, Orlova Roshcha, Gatchina, Russia
[6] Belozersky Institute of Physico-Chemical Biology, Lomonosov Moscow State University, Moscow, Russia
[7] These authors contributed equally to this work
*Correspondence: stanishneva-konovalova@mail.bio.msu.ru

Supplementary information

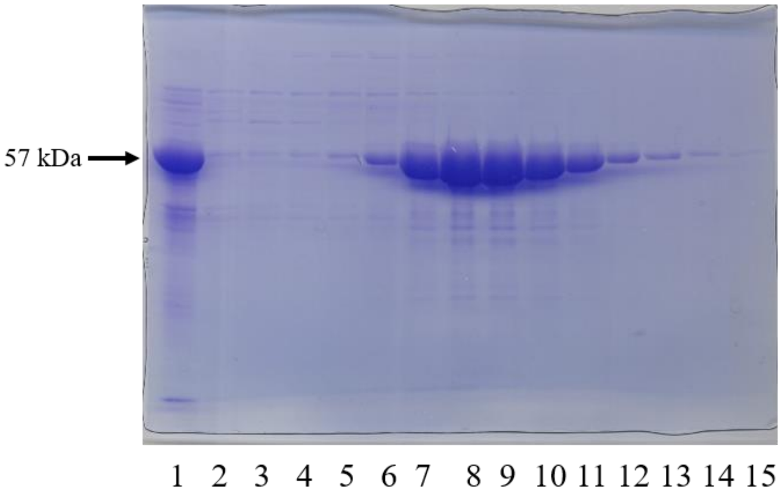

Figure S1. SDS-PAGE of samples obtained at the last stage of GroEL purification on DEAE-Sephacel. 1 - GroEL standard solution; 2-15 - fractions collected during second chromatography. The arrow shows the band corresponding to the prion protein.

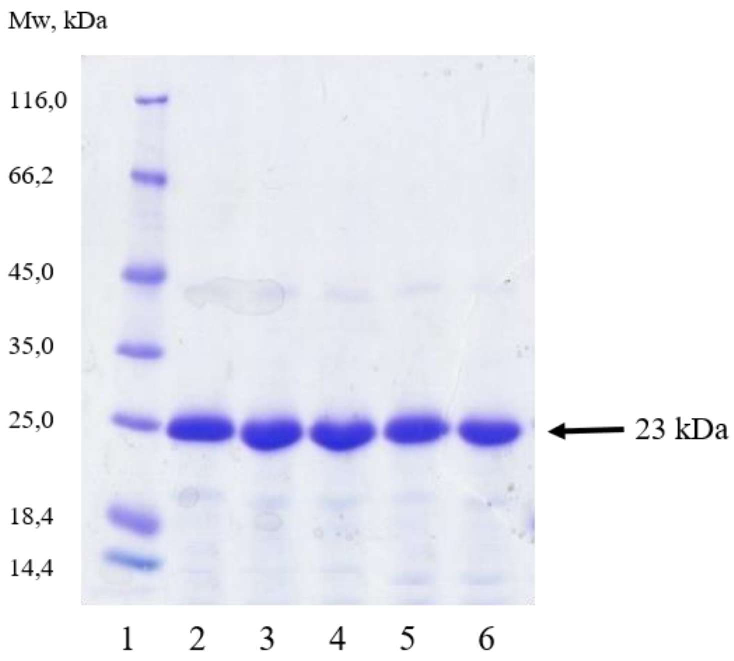

Figure S2. SDS-PAGE of samples obtained during the purification of the prion protein carried out on the Ni2+-Chelating Sepharose fast flow. 1 - protein molecular weight marker; 2-6 - fractions obtained by affinity chromatography. Molecular weight markers are indicated on the left. The arrow shows the band corresponding to the prion protein.

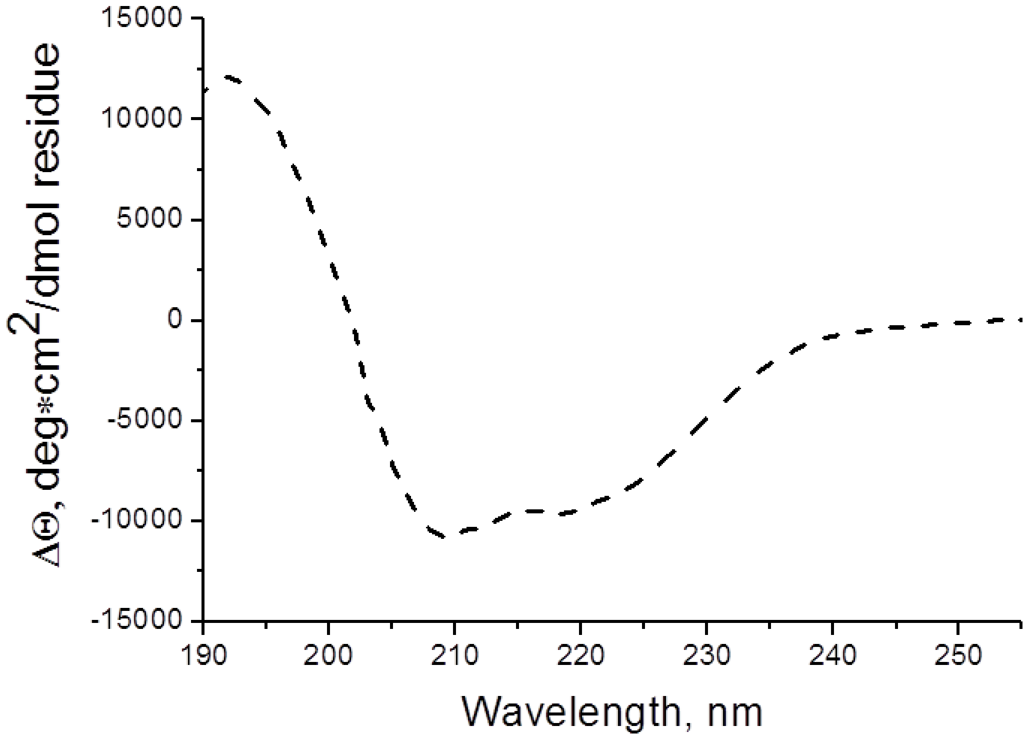

Figure S3. Far-UV CD spectra of the recombinant VRQ (V136, R154, Q171) variant of ovine PrP (23–234) at 20°C. [θ]MRW, mean residue ellipticity (deg×cm2/dmol of residue).

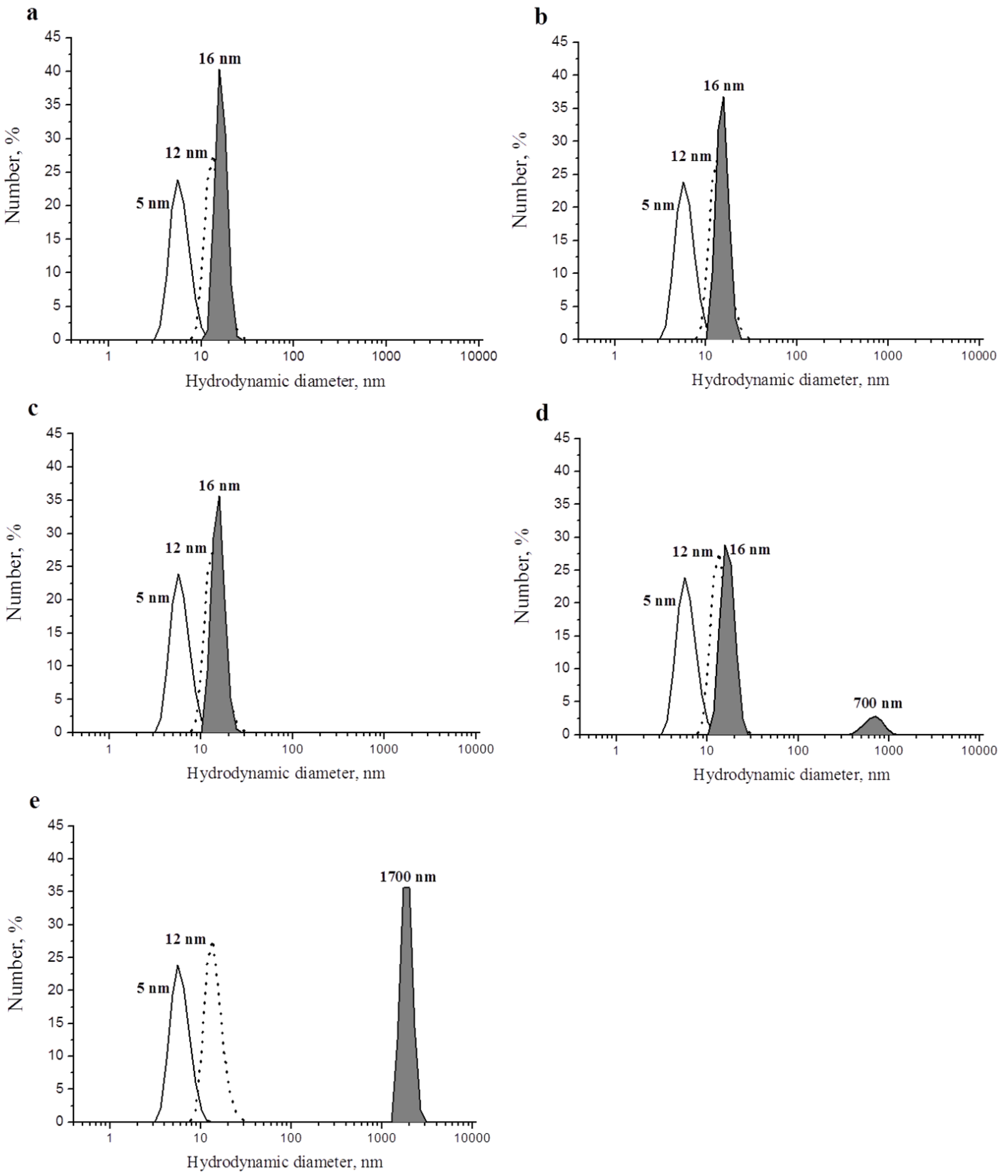

Figure S4. Hydrodynamic diameter of particles detected after co-incubation of GroEL (8 µM) with PrP monomers (16 µM) in 50 mM Tris-HCl buffer, pH 7.5 containing 1 mM EDTA. The populations of particles that are complexes are in gray, and the control of individual chaperonin complexes (dotted line) and PrP monomers (solid line) are shown separately on the plots. a) 0 min of co-incubation; b) 10 min of co-incubation; c) 30 min of co-incubation; d) 50 min of co-incubation; e) 120 min of co-incubation

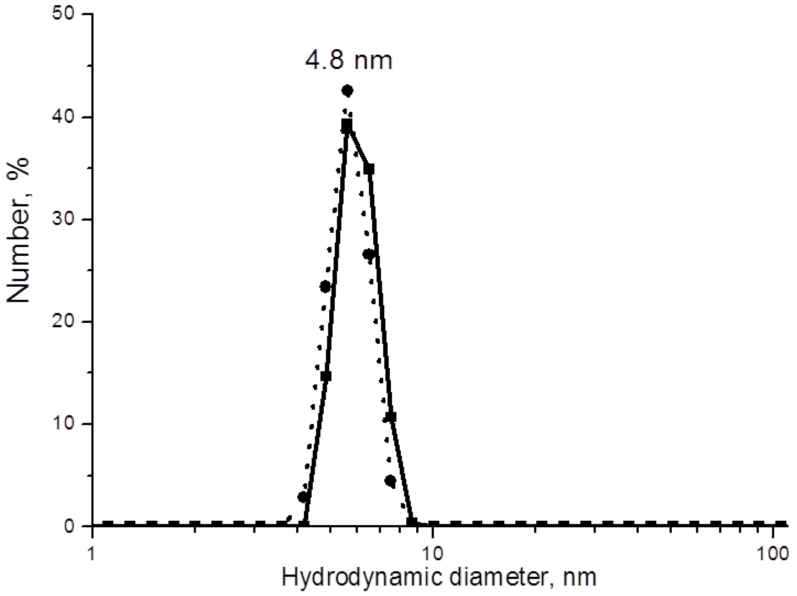

Figure S5. Hydrodynamic diameter of PrP molecules in 50 mM Tris-HCl buffer, pH 7.5 containing 1 mM EDTA before (solid line) and after (dotted line) incubation at 21°C for 2 hrs.

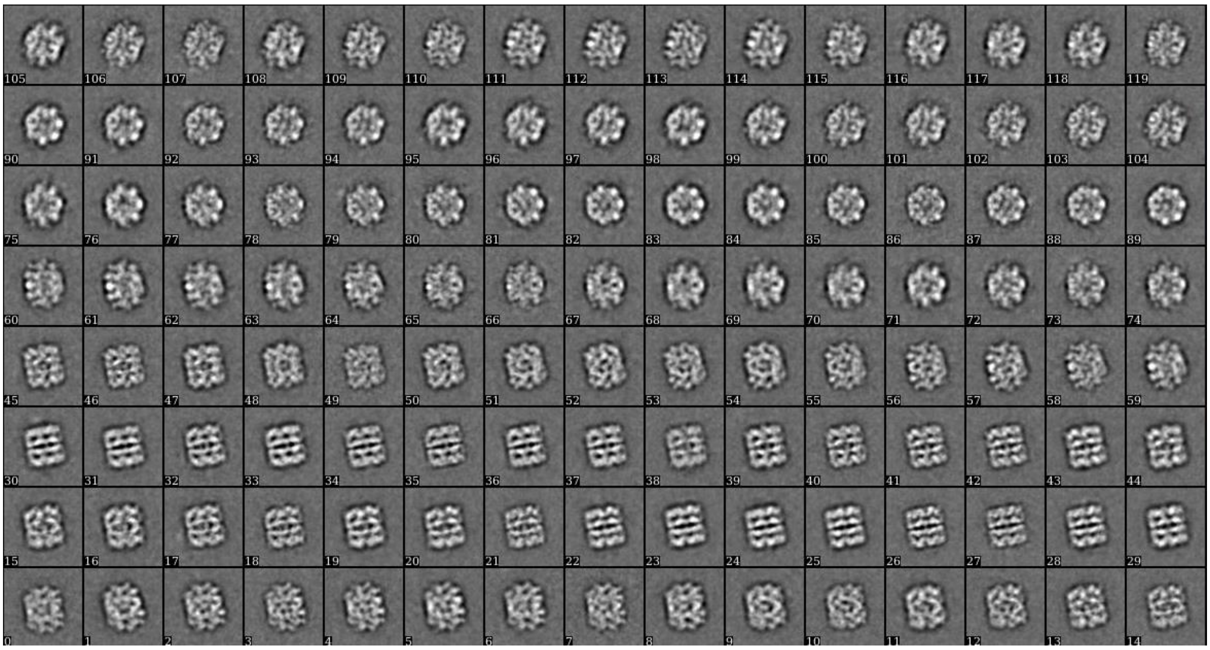

Figure S6. ISAC 2d class averages. Top view class averages #75-104 (2nd to 4th rows from the top) exhibit an additional density in the central cavity.

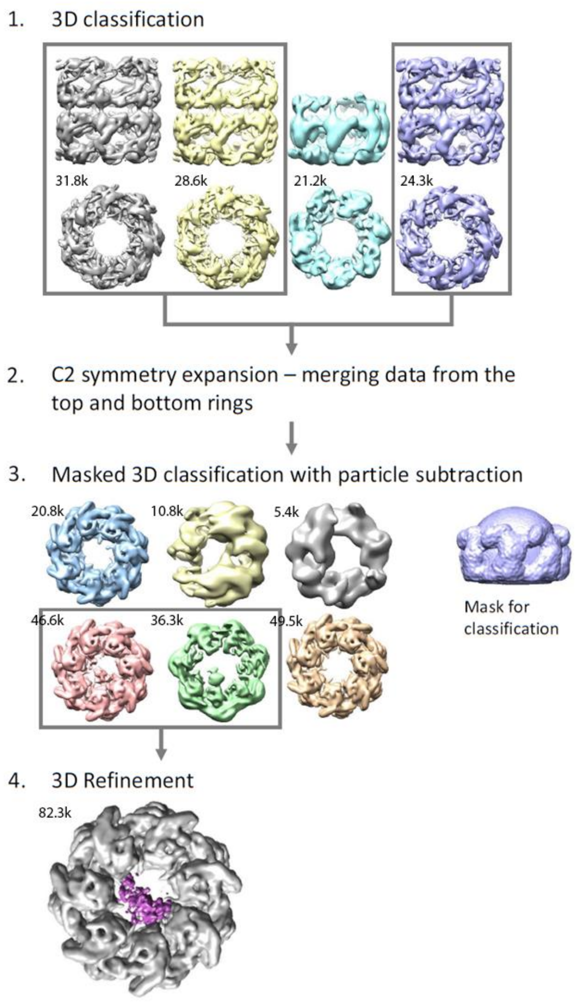

Figure S7. Single particle processing workflow. Number of particles in each class is indicated.

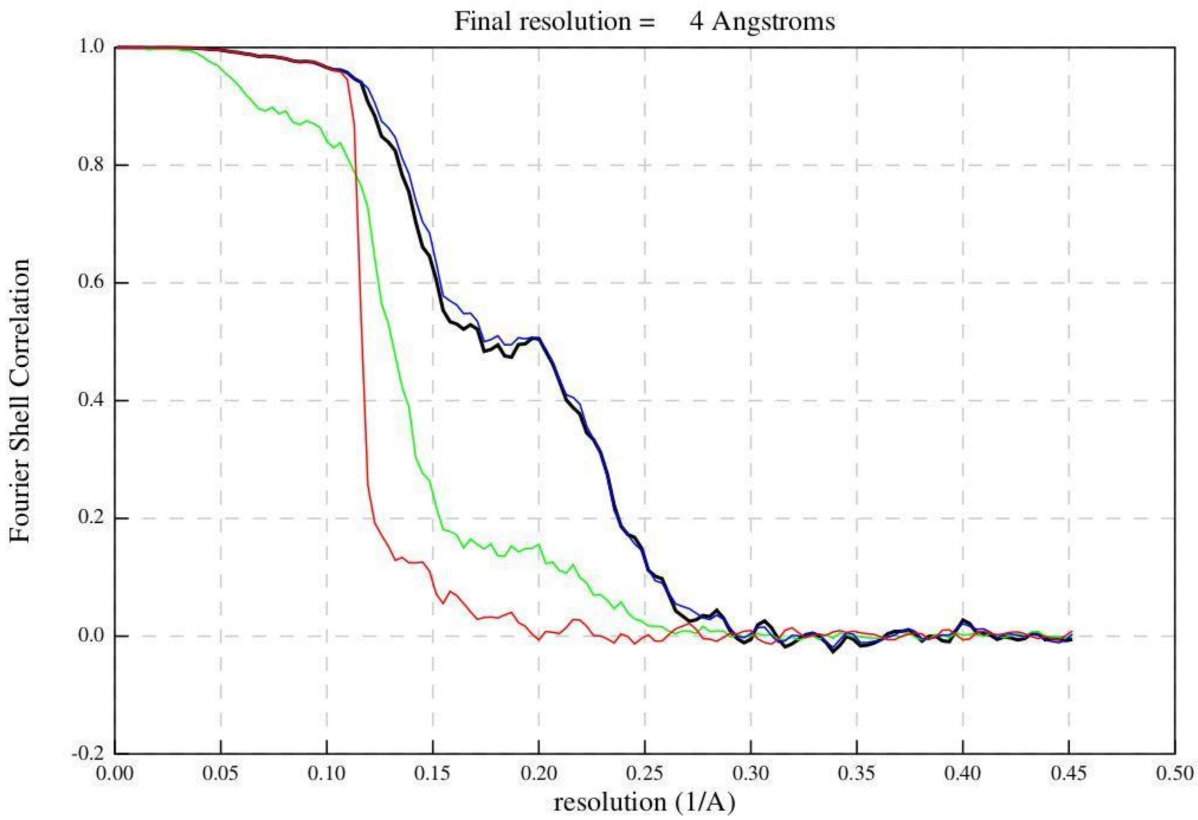

Figure S8. FSC curves for the symmetry-expanded reconstruction provide the 4A resolution based on 0.143 criterion. (Green – unmasked half-maps, blue – masked half-maps, red – phase randomized masked half-maps, black – corrected).

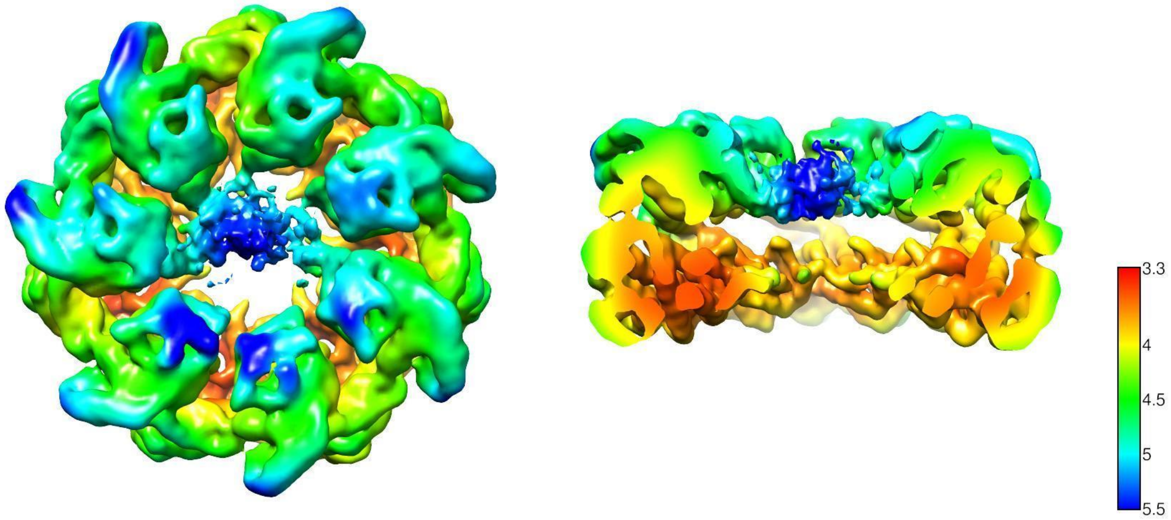

Figure S9. GroEL-PrP cryo-EM local resolution map.

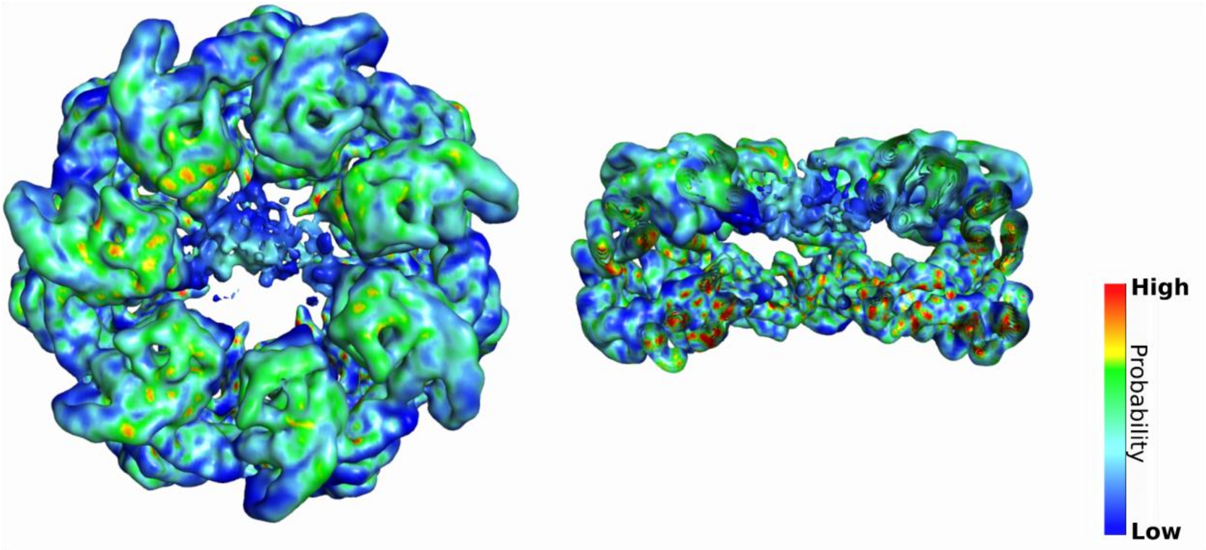

Figure S10. Heavy atoms distribution combined for both GroEL-PrP(N) and GroEL-PrP(C) MD simulations projected on the Cryo-EM density map.

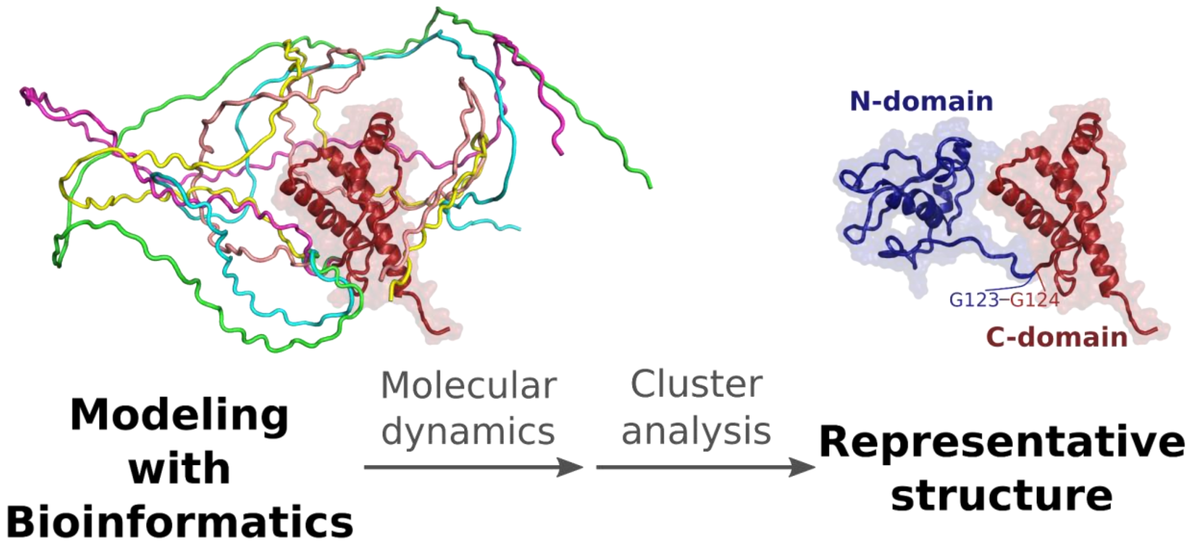

Figure S11. Workflow for the full-length PrP model creation. Red cartoon+surface is the representation of the globular C-domain, varicoloured coils on the left represent top-scored solutions of de novo modeling for the N-domain in Alpfafold II [Jumper J. et al. Highly accurate protein structure prediction with AlphaFold //Nature. – 2021. – T. 596. – №. 7873. – C. 583-589.], blue cartoon+surface on the right represents the common structure of the compact N-domain obtained after subsequent molecular dynamics and a cluster analysis of its conformations. G123-G124 are the chosen residues to separate the N- and C-domains in the representation.

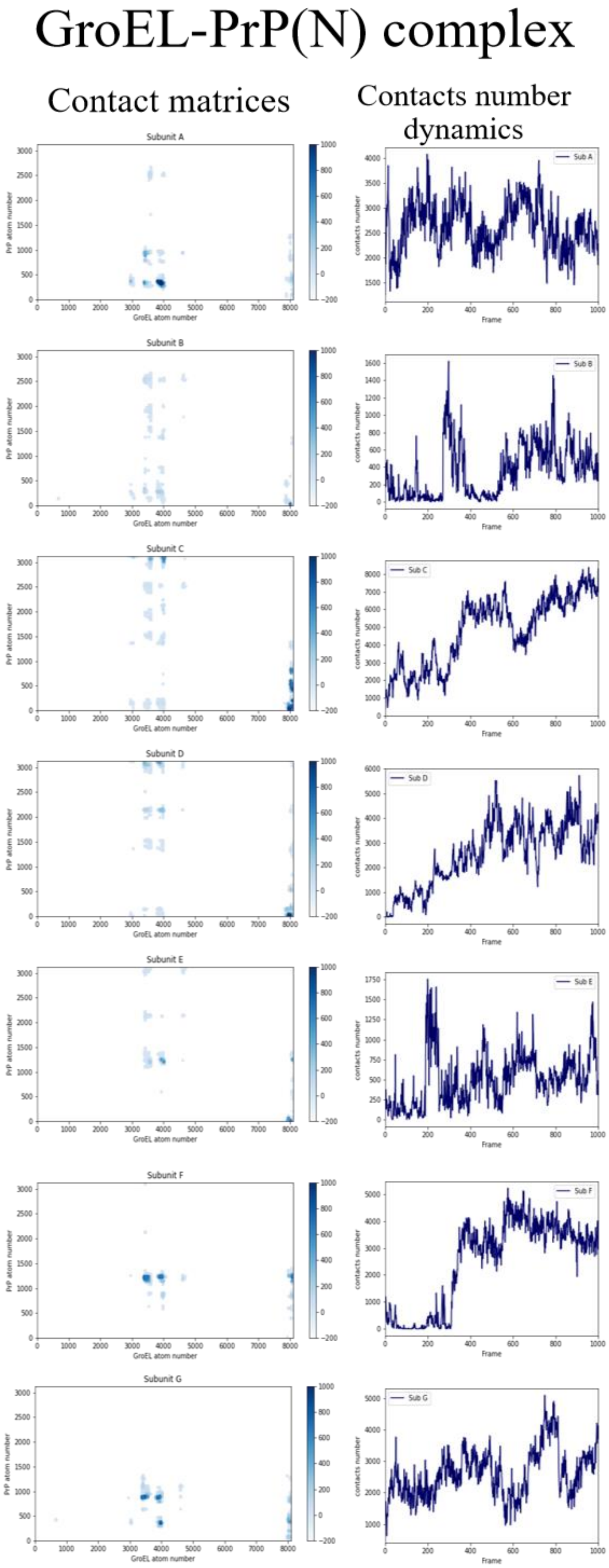

Figure S12. Contact matrices and dynamics of the number of contacts of the GroEL-PrP(N) complex by GroEL subunits.

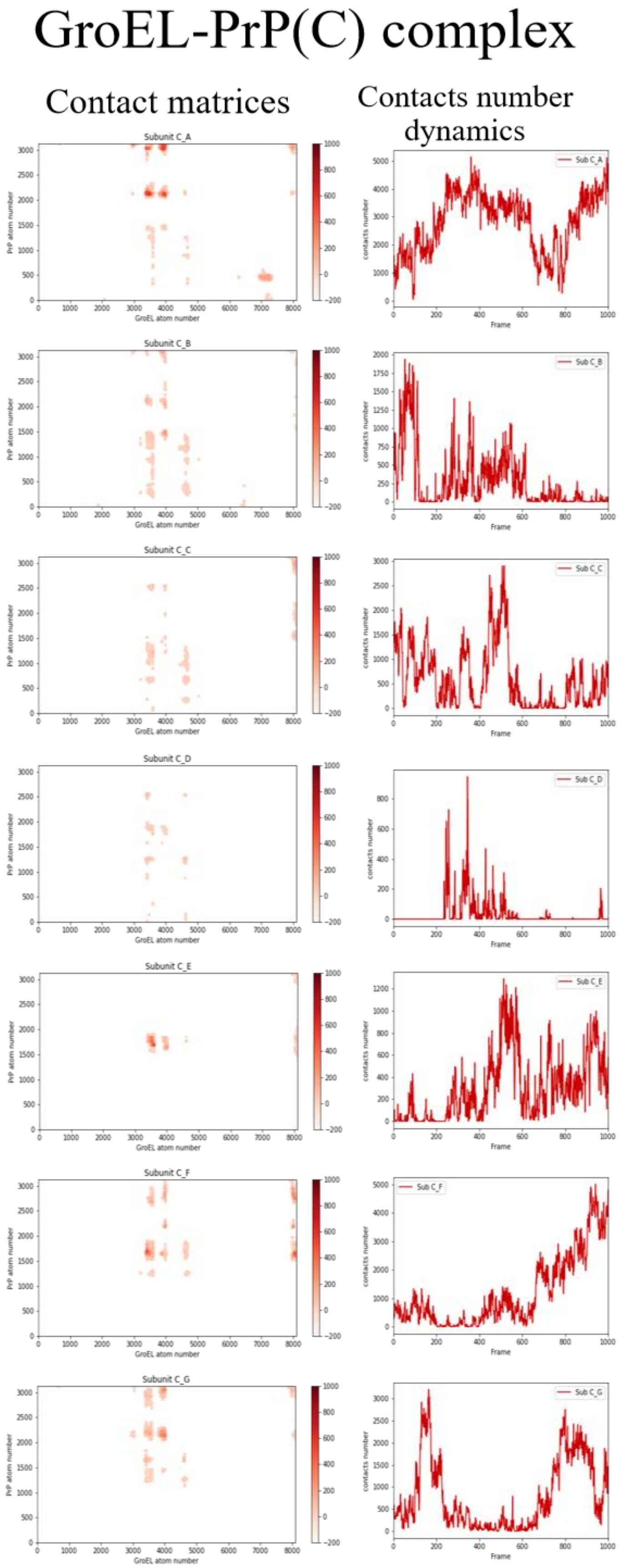

Figure S13. Contact matrices and dynamics of the number of contacts of the GroEL-PrP(C) complex by GroEL subunits.

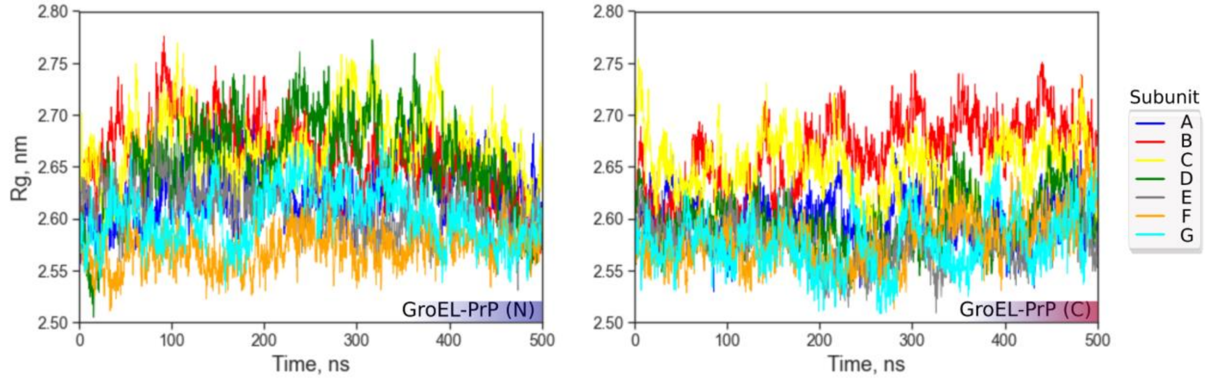

Figure S14. GroEL subunits mobility in terms of the radius of gyration (Rg): GroEL-PrP(N) trajectory (left); GroEL-PrP(C) trajectory (right). Rg of each subunit was calculated separately.

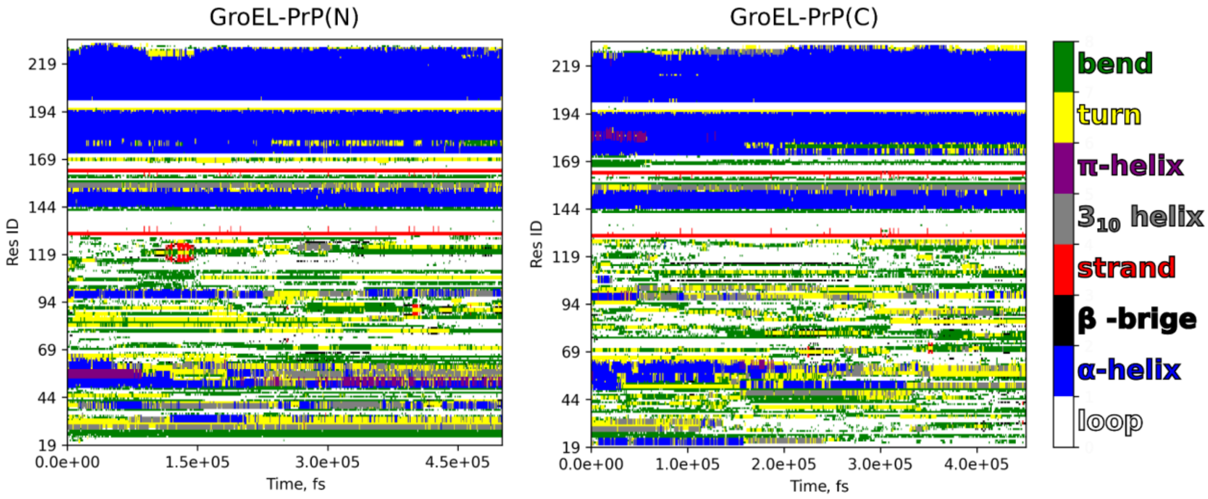

Figure S15. Dynamics of the PrP secondary structure along the GroEL-PrP(N) (left) and GroEL-PrP(C) (right) trajectories.

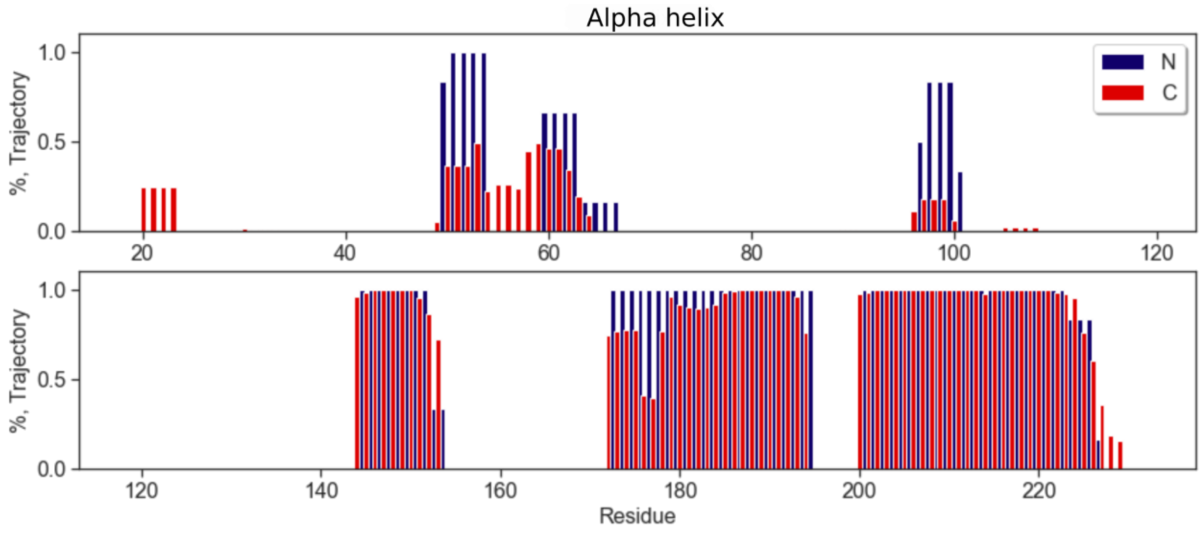

Figure S16. Presence of alpha-helices in the PrP structure across the calculated trajectory.

Video S1: ISAC 2d classes showing the mobility of the PrP inside the GroEL cavity.

Video S2: The course of MD trajectories with the GroEL-PrP(N) complex. Proteins are shown with tube representation using VMD program (version 1.9.4a12): grey - GroEL, blue - PrP N-terminal domain, red - PrP C-terminal domain. Only the Cα atoms of both proteins are shown for clarity. The trajectory is shown with a 250 ps timestep, with a smoothing window size of 10 for GroEL and 5 for PrP.

Video S3: The course of MD trajectories with the GroEL-PrP(C) complex. Proteins are shown with tube representation using the VMD program (version 1.9.4a12): grey - GroEL, blue - PrP N-terminal domain, red - PrP C-terminal domain. Only the Cα atoms of both proteins are shown for clarity. The trajectory is shown with a 250 ps timestep, with a smoothing window size of 10 for GroEL and 5 for PrP.

Videos S1-S3 are available in the Zenedo archive (10.5281/zenodo.5590094).

Table S1: Data collection and processing statistics

Grid type	*Copper Quantifoil R1.2/1.3*
Microscope/Detector	*Titan Krios/Falcon II*
Accelerating voltage	*300 kV*
Total dose	*100 e-/Å2*
Number of frames	*25*
Defocus	*1.0–2.6 mkm*
Pixel size	*1.107 Å*
Number of micrographs	*1621*
Total particles picked	*106k*
Number of particles in final reconstruction (after symmetry expansion)	*82.3k*
Map resolution (0.143 FSC)	*4.0 Å*
EMD	*EMD-13762*
